# Identification and characterization of human cytomegalovirus-encoded circular RNAs

**DOI:** 10.3389/fcimb.2022.980974

**Published:** 2022-11-14

**Authors:** Jingui Deng, Qing Wang, Jing Zhang, Yanping Ma, Ying Qi, Zhongyang Liu, Yibo Li, Qiang Ruan, Yujing Huang

**Affiliations:** ^1^ Department of Microorganism Laboratory, Shenyang Center for Disease Control and Prevention, Shenyang, China; ^2^ Virology Laboratory, Shengjing Hospital of China Medical University, Shenyang, China; ^3^ Department of Pediatrics, Shengjing Hospital of China Medical University, Shenyang, China; ^4^ Department of Obstetrics and Gynecology, Shengjing Hospital of China Medical University, Shenyang, China; ^5^ Department of Obstetrics and Gynecology, Central Hospital Affiliated to Shenyang Medical College, Shenyang, China

**Keywords:** human cytomegalovirus (HCMV), noncoding RNA, circular RNA (circRNA), sequence, function

## Abstract

Circular RNA (circRNA) exists extensively and plays essential roles in serving as microRNA (miRNA) or protein sponges and protein scaffolding in many organisms. However, the profiles and potential functions of the virus-encoded circRNA, including human cytomegalovirus (HCMV)-encoded circular RNAs, remain unclear. In the present study, HCMV-encoded circRNAs profile in human embryonic lung fibroblasts (HELF) with lytic infection was investigated using RNA deep sequencing and bioinformatics analysis. In total, 629 HCMV-encoded circRNAs were identified with various expression patterns in our results. The full sequences and alternative splicings of circUS12, circUL55, and circUL89 were verified by reverse transcriptase-PCR (RT-PCR) with divergent primers followed and Sanger sequencing. Transcription of circUL89 was validated by Northern blot. The HCMV-encoded circRNA-miRNA network analyses revealed the potential function of HCMV-encoded circRNAs during HCMV infection in HELFs. Collectively, HCMV infection deduced abundant HCMV-associated circRNAs during infection, and the HCMV-encoded circRNAs might play important roles in benefiting HCMV infection.

## Introduction

Human cytomegalovirus (HCMV) is a ubiquitous beta-herpesvirus that can infect a range of essential organs, including the brain, heart, lungs, liver, kidney, blood, bone marrow, and salivary gland (Cobbs et al., 2002; Cheeran et al., 2009; Abdel-Latif and Sugo, 2010). Although it is asymptomatic in immunologically healthy populations, HCMV infections can cause life-threatening diseases in congenitally infected newborns and immunocompromised patients such as transplant recipients and AIDS patients (Einsele and Hebart, 1999; Khoshnevis and Tyring, 2002; Whitman et al., 2005; Crough and Khanna, 2009). HCMV has a double-stranded genome of approximately 230-kb, which encodes more than 165 proteins (Murphy et al., 2003; Dolan et al., 2004; Ma et al., 2011b). Also, the HCMV genome has a variety of transcripts, including sense transcript (Murphy et al., 2003), antisense transcript (Ma et al., 2011a), splice transcript (Gao et al., 2015a), and many noncoding RNAs (ncRNA) transcripts (Jiang et al., 2017). Attributing to the complex transcriptome of HCMV, the pathogenesis of HCMV still remains unclear.

Circular RNAs (circRNAs) are a kind of ncRNA characteristic of a covalently closed loop without a polyadenylated tail by joining the 3’ donor site to the 5’ acceptor site, which is called back-splicing (Lasda and Parker, 2014). This feature benefits circRNAs more prone to resist exonucleases (such as RNase R) than their linear counterparts (Houseley et al., 2006; Wilusz and Sharp, 2013). CircRNAs were firstly found in plant viruses in 1976 (Sanger et al., 1976). However, being considered the byproducts of formal transcription, circRNAs have been out of the spotlight for decades (Nigro et al., 1991; Cocquerelle et al., 1993; Zaphiropoulos, 1996). In recent years, along with the development of sequencing technology and bioinformatics methods, more and more circRNAs have been discovered in many species (Salzman et al., 2012; Hentze and Preiss, 2013; Jeck et al., 2013; Memczak et al., 2013).

Various functions have been identified for circRNAs, such as, to serve as microRNA (miRNA) sponges as a competitor with the linear counterpart gene (Zheng et al., 2016; Fan et al., 2021; Han et al., 2021; Yu et al., 2022; Chen et al., 2022a), or as a scaffold for RNA binding proteins (Wu et al., 2021; Mo et al., 2021; Chen et al., 2022a). More recently, circRNAs have been found to be the potential to translate proteins (Pamudurti et al., 2017; Wesselhoeft et al., 2018; Gao et al., 2021; He et al., 2021; Chen et al., 2022b; Wen et al., 2022). More recently, circRNAs have shown their potential functions as vaccines or antisense-circRNA against severe acute respiratory syndrome coronavirus 2 (SARS-CoV-2) (Pfafenrot et al., 2021; Qu et al., 2022; Szabó et al., 2022).

In our previous work, we found that HCMV lytic infection enhanced the production of host circSP100, which might facilitate virus proliferation *via* serving as DNA-dependent protein kinase (DNA-PK) sponge (Deng et al., 2021). Recently, more and more virus-encoded circRNAs were demonstrated with diverse functions. For example, it was reported that hepatitis B virus (HBV)-encoded HBV_circ_1 could promote hepatocellular carcinoma by interacting with cyclin-dependent kinase 1 (Zhu et al., 2021). CircE7 encoded by human papillomavirus type 16 (HPV16) E7 is essential for the expression of oncoprotein E7 and is associated with tumor xenografts (Zhao et al., 2019). However, little is known about the transcription and function of HCMV-encoded circRNAs.

In this study, the HCMV-encoded circRNA profile was investigated in human embryonic lung fibroblasts (HELFs) with lytic infection. Viral circRNAs with high transcriptional levels were validated by reverse transcription-polymerase chain reaction (RT-PCR) and Northern blot. Subsequently, putative target miRNAs of validated HCMV-encoded circRNAs were predicted and further analyzed to speculate the functions of viral circRNAs in infection.

## Materials and methods

### Cell preparation

HELF cells were obtained from the Shanghai Institute for Biological Sciences, Chinese Academy of Sciences (CAS). The HELF cells were cultured in minimum essential medium (MEM) (HyClone) supplemented with 10% fetal bovine serum (FBS) (Gibco), 100 units/mL penicillin, and 100 μg/mL streptomycin at 37°C with an atmosphere of 5% CO_2_.

### Virus preparation and infection

HCMV low-passage strain Han labeled with a green fluorescent protein (GFP) was used for virus preparation. Viral stocks were generated following standard ultracentrifugation procedures described previously (Zhao et al., 2016).

HELFs were infected with HCMV at a multiplicity of infection (MOI) of 3. After 24 hours of incubation, the culture medium was replaced with MEM containing 2% FBS, 100 units/mL penicillin, and 100 μg/mL streptomycin. Meantime, PBS-treated HELFs were prepared as a mock-infected control. Infection was confirmed at 72 hours post infection (hpi) (**Supplementary Figure S1**). The HCMV-infected and mock-infected HELFs were harvested.

### RNA preparation

According to the manufacturer’s protocol, total RNAs were extracted using the TRIzol Reagent (Thermo Fisher). The integrity of the total RNAs was evaluated using an Agilent 2100 Bioanalyzer (Agilent Technologies), and the concentration of the RNAs was measured using the NanoDrop 1000 (Thermo Scientific). Ribo-Zero rRNA Removal Kits (Epicentre) were used to remove ribosomal RNA from the total RNAs. Then, the rRNA-depleted samples were treated with RNase R treatment (Epicentre) to digest linear RNAs according to the manufacturer’s instructions.

### Libraries construction and RNA sequencing

The treated RNAs were fragmented and reversely transcripted into cDNA using SuperScript™ II Reverse Transcriptase (ThermoFisher) according to its protocol. RNA libraries were constructed using the TruSeq RNA LT Sample Prep Kit v2 (Illumina). Paired-end RNA sequencing was performed on an IlluminaHiSeq 2500 at Biotechnology Co. Ltd., Shanghai, China. The RNA sequencing data were deposited in the Gene Expression Omnibus database under accession number GSE138836.

### Prediction and analysis of HCMV-encoded circRNAs

As previously described (Deng et al., 2021), clean reads were obtained using Seqtk (https://github.com/lh3/seqtk). The filtered reads were first mapped to the human reference genome (GRCH37/HG19). The remaining unmapped reads were then mapped to the HCMV genome (NC_006273.2) with BWA-MEM (version 0.7.13) (Li, 2013). The mapped broken-paired reads were analyzed by CIRI (version 1.1) to identify HCMV-encoded circRNAs with default parameters, including junction reads with paired-end mapping (PEM) and GT-AG splicing signals (Gao et al., 2015b). The length of circRNA was predicted by calculating the length of HCMV genome between the 3’ donor site and the 5’ acceptor site.

Predicted circRNAs with more than two backspliced junctions (BSJ) reads were identified as candidate HCMV-encoded circRNAs. Candidate circRNAs with BSJ reads of more than 50 were determined as prominent circular transcripts of HCMV in our research. The expression of the circRNA candidates was estimated by spliced reads per billion mappings (Li et al., 2015a).

### Design of divergent primers (DPs)

**Figure 1:**
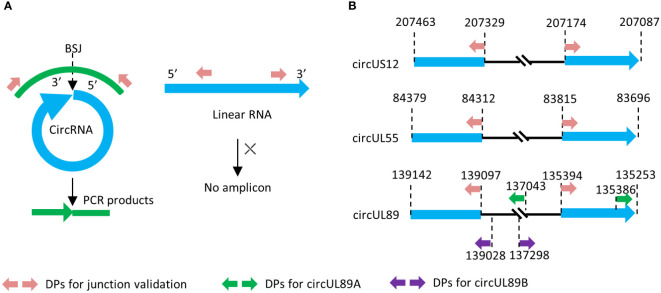
Principle of divergent primer design and amplification. **(A)** Principle of divergent primer in amplification of circRNA and linear counterpart. **(B)** Genome locations of the divergent primes in our research.

Divergent primers targeting the BSJ sequences of HCMV-encoded circRNAs (circUS12, circUL55, and circUL89) were designed with Primer Premier 6.00 (PREMIER Biosoft International). Sequences covering BSJ and flanking regions were obtained from RNA sequencing data and subjected to primer design. The amplicons should contain their junction sites and be preferably 200 ~300 bp in size (**Figures 1A, B**).

To gain the entire sequence of circUL89 transcript, two other pairs of divergent primers were designed with products of about 2000bp in length. As shown in **Figure 1B**, the terminals of the two products overlapped each other, and the transcript of circUL89 could be achieved by splicing and connecting the sequences of the products. Primer sets mentioned above are listed in **Supplementary Table S1**.

### Reverse transcription, inverse RT-PCR and sequencing

Total RNA from HCMV-infected HELFs was isolated using TRIzol reagent (Life Technologies). For RNase R treatment, 4μg of total RNA was incubated with RNase R (5 units/μg RNA) or RNase-free water as a control for 30 minutes at 37 ˚C. RT-PCR analysis was performed with PrimeScript RT-PCR Kit (TaKaRa) according to the manufacturer’s protocol. Convergent primers of glyceraldehyde 3-phosphate dehydrogenase (GAPDH, NM_002046.7) and HCMV UL123 were used as controls.

PCR products were detected by electrophoresis on 1.5% agarose gel containing ethidium bromide and visualized under UV light. PCR products with expected sizes were purified using Wizard Plus SV Minipreps DNA Purification System (Promega) and cloned into pCR2.1vector using TA Cloning Kit (Thermo Fisher). Sanger sequencing was performed in ThermoFisher Scientific Co. Ltd., Shanghai, China.

### Sequence homology analysis

For each sequence obtained from the TA clone, about 90bp sections around the 5’ and 3’ junction sites were analyzed using DNAClub and compared using BioEdit to identify homologous sequences in the HCMV genome (NC_006273.2).

### Northern blot

DIG-labeled sense and antisense probes targeting circUL89 BSJ site were designed and synthesized with DIG Northern Starter Kit (Roche) according to instructions. Antisense probes targeting β-actin (NM_001101) were also synthesized to detect β-actin, which were used as the loading controls. Primers for generating these probes are listed in **Supplementary Table S1**.

A total of 20 μg RNA for each detection was separated on agarose containing 2% formaldehyde and transferred to IMMOBILON-NY+ nylon membranes (MILLIPORE), and the membrane was rinsed and baked as previously (Deng et al., 2021). Hybridization was performed with DIG-labeled probes at 60°C overnight. After incubation, the membrane was washed and incubated with anti-DIG-AP. Detection was performed as instructed, and photos were captured by Bio-Rad molecular imager chemiDox XRS with ImageLab software (Bio-Rad).

### CircRNA-miRNA network construction

To gain insights into the relationship between miRNAs and HCMV circRNAs, putative targeted miRNAs of the selected HCMV-encoded circRNAs, including circUS12, circUL89, and circUL55, were predicted by miRanda (Enright et al., 2003). MiRNAs with maximum scores ≥140 and maximum energy≤ −20 kcal/mol were determined as candidate miRNA targets of viral circRNAs.

The circRNA-miRNA network was constructed using Cytoscape (v3.4.0) (Friedländer et al., 2012). To assess the potential biological function of circUS12, we performed a cluster analysis of their putative target miRNAs for Gene Ontology (GO, http://www.geneontology.org/).

## Results

### HCMV produces circRNAs during lytic infection

**Figure 2:**
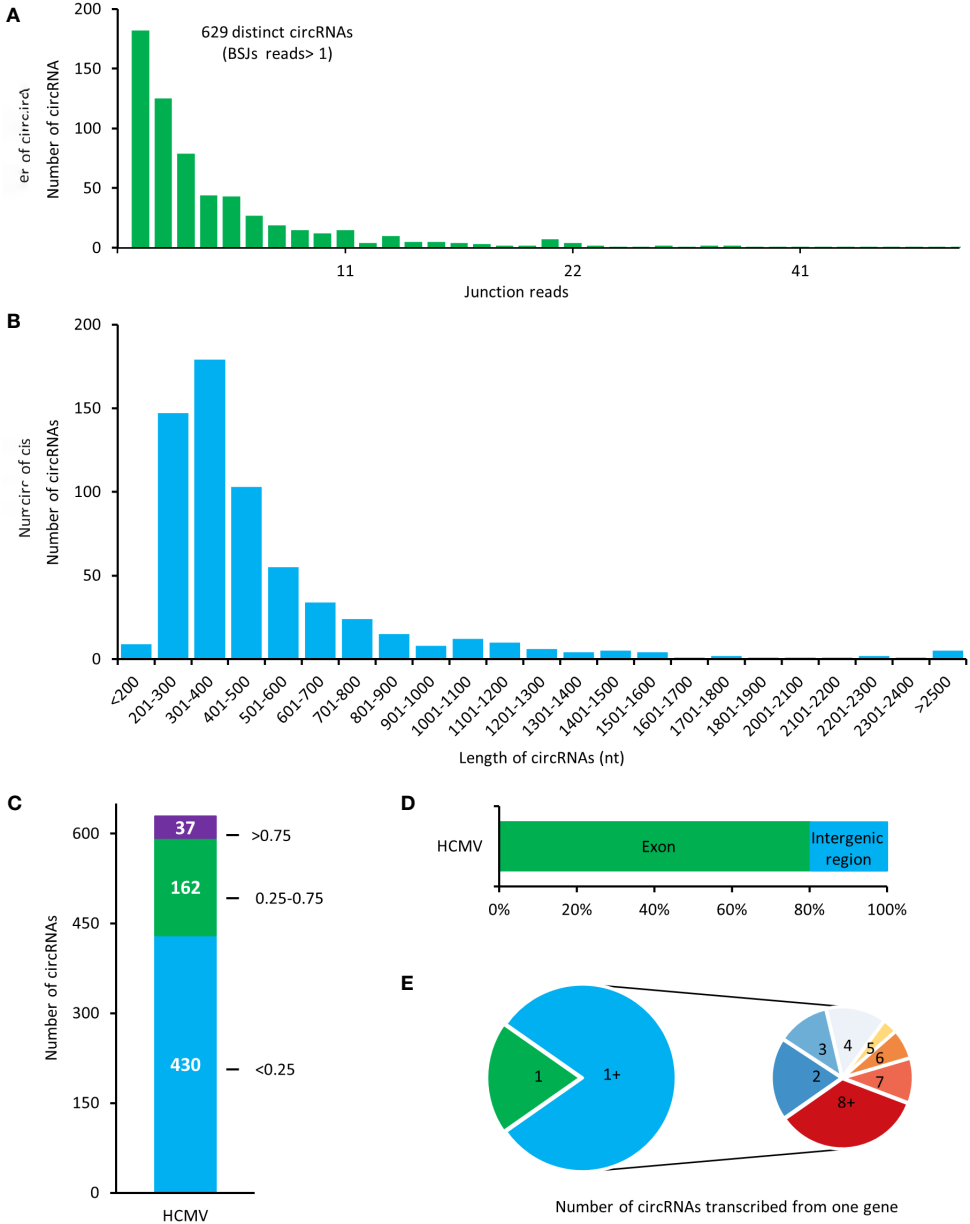
Expression patterns of HCMV-encoded circRNAs in HELF cells. **(A)** The total numbers of HCMV-encoded circRNAs and BSJs read identified in this study. **(B)** Length distribution of HCMV-encoded circRNAs. **(C)** Distribution of spliced reads per billion mappings (SRPBM) of HCMV-encoded circRNAs. **(D)** Classification of circular HCMV-encoded RNAs based on the genomic origin. **(E)** The number of viral circRNAs transcribed from one gene.

Total RNAs of HELFs with HCMV lytic infection were treated with RNase R before RNA sequencing. The filtered reads were firstly mapped to the human reference genome, and the remaining unmapped reads were then mapped to the HCMV genome. A total number of 629 potential HCMV-encoded circRNAs were identified in our results (**Figure 2A**). In our results, most of the viral circRNA (623/629) has a low value of BSJ reads less than 50. By calculating the transcription length of circRNA in our results, it was found that most of the circRNAs were distributed in the range of 200-600 nt, and the median length of the circRNAs was ~500 nt (**Figure 2B**).

Values of spliced reads per billion mappings (SRPBM) for each viral circRNA were calculated to address the relative expression levels of HCMV-encoded circRNAs. The SRPBM of HCMV-encoded circRNAs ranged from 0.086 to 10.569 (**Figure 2C**). Totally, 37 circRNAs presented higher SRPBM values of more than 0.75.

By comparing the genomic origin sites of viral circRNAs, it was found that about 80% of viral circRNAs were transcribed from the exonic region (termed exonic circRNA). In contrast, the rest circRNAs were mainly transcribed from the intergenic region (**Figure 2D**). Furthermore, mapping the circRNAs to their host genes found that the 504 exonic cricRNAs detected in our study were transcribed from 77 HCMV genes (**Figure 2E**). Similar to the mechanism of alternative splicing in mRNA transcriptions, the HCMV gene could produce circRNAs with various splicing and junction formations in HELF cells.

The abundance of candidate viral circRNAs was ranked according to their BSJ reads. Detailed information on the top-ranked 20 HCMV-encoded circRNAs in HELFs is listed in **Table 1**. HCMV circUS12, circUL150A, circUL89, and circUL55 were the top-ranged circRNAs. Subsequently, prominent transcripts of circUS12 (NC_006273.2:207087|207463), circUL89 (NC_006273.2:135253|139142), and circUL55 (NC_006273.2:83696|84379) were selected for further investigation.

**Table 1:** The top 20 HCMV-encoded circRNAs in HELFs.

Gene ID	start	end	length	Junction reads	circRNA type	The function of the host gene
US12	207087	207463	377	246	exon	immune regulation (Fielding et al., 2017)
UL89	135253	139142	3890	100	exon	Viral genome packing (Neuber et al., 2017; Theiß et al., 2019)
UL150A	187194	188486	1293	78	exon	HCMV infection (Gatherer et al., 2011)
UL55	83696	84379	684	68	exon	virus entry, cell-to-cell spread (Isaacson and Compton, 2009)
UL13	19816	20271	456	62	exon	unclear
UL150A	184899	185274	376	54	exon	HCMV infection (Gatherer et al., 2011)
UL36	48791	49251	461	44	exon	anti-apoptosis (Skaletskaya et al., 2001)
UL36	49201	49618	418	42	exon	anti-apoptosis (Skaletskaya et al., 2001)
US33A	230646	231085	440	41	exon	unclear
US22	217299	217609	311	40	exon	tegument components (Adair et al., 2002)
N/A	32484	32766	283	37	intergenic region	–
N/A	217734	218041	308	36	intergenic region	–
UL5	14529	14725	197	36	exon	Viral replication (Gonzalez-Perez et al., 2021)
UL150A	193835	194044	210	33	exon	HCMV infection (Gatherer et al., 2011)
UL36	49577	50050	474	33	exon	anti-apoptosis (Skaletskaya et al., 2001)
UL55	83594	83840	247	32	exon	virus entry, cell-to-cell spread (Isaacson and Compton, 2009)
N/A	27477	27885	409	31	intergenic region	–
UL34	45826	46126	301	31	exon	Viral replication (Rana and Biegalke, 2014)
US33A	230678	231066	389	30	exon	unclear

### HCMV circUS12 possesses multiple isoforms derived by alternative splicing

To address the circular characteristic of circUS12, RNA extracted from HCMV-infected HELFs was treated with RNase R, reverse transcribed, and amplified using divergent primers targeting the circUS12 BSJ sequence. In contrast to convergent primers, divergent primers are characteristic of facing away from each other on the linear RNA. Theoretically, divergent primers can only amplify the circRNAs, not the linear RNAs with the same sequence (Panda and Gorospe, 2018). In our study, RT-PCR analysis using the circUS12 primer set (the divergent primers) allows direct detection of circular US12 RNA. The anticipated size of products containing BSJ of the circUS12 was 226nt. Meantime, linear UL123 and GAPDH were detected as controls to assess the efficiency of RNase R treatment.

**Figure 3:**
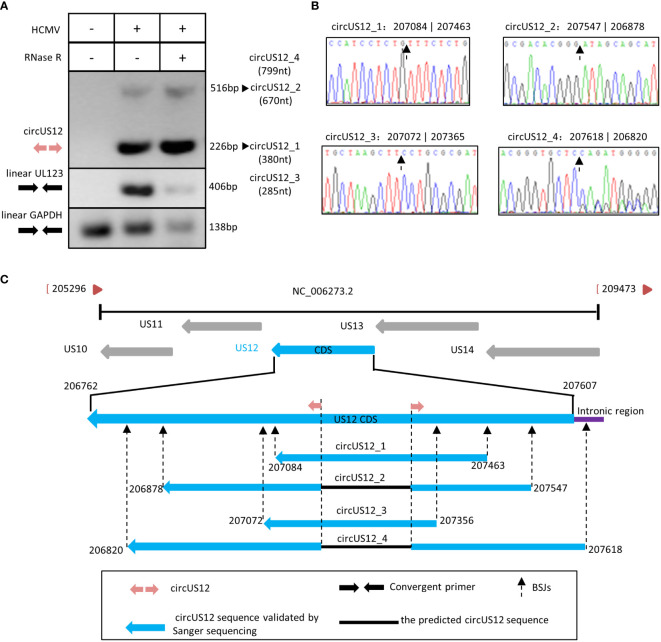
Validation and characterization of HCMV circUS12. **(A)** Inverse RT-PCR products of circUS12 with BSJs validation primer set in **Figure 1**. The 226 bp band, which was amplified from circUS12_1 (380 nt), was the predicted amplicon of the primer. Interestingly, the BSJ primer achieved a 516 bp band, which was a part of circUS12_2 (799nt). Linear GAPDH and linear UL123 were amplified as RNase R-resistant control. **(B)** Sanger sequencing result of circUS12_1, circUS12_2, circUS12_3, and circUS12_4 BSJs. The BSJ of circUS12_1 was consistent with RNA-seq. The other three circUS12 BSJ that hadn’t been demonstrated in RNA-seq, and were revealed in Sanger sequencing. **(C)** Four circUS12 isoforms mapping to HCMV US12 gene.

By amplification, we obtained two different products with different lengths, 226nt, and 516nt, respectively (**Figure 3A**). All products with all lengths were cloned and sequenced. Sequencing analysis results suggested the main amplicons were derived from two isoforms of circUS12, circUS12_1, and circUS12_2, respectively (**Figure 3B**). The BSJ sequence of circUS12_1 was the primary product and was consistent with our BSJ predicted results. CircUS12_2 was expected to be a viral circRNA with 670nt in length. Moreover, we found some products contained a repeat sequence of circUS12_1 BSJ. This might be due to a “rolling replication**”** during reverse transcription (You et al., 2015; Barrett et al., 2015). By analyzing sequences obtained from rolling replication, the full length of circUS12_1 was achieved, which was 380nt in length (**Figure 3C**). Thus, our study obtained only parts of the circUS12_2 sequence (**Figure 3C**), accounting for the relatively short extension time in “rolling replication.”

Additionally, we got two more isoforms (circUS12_3 and circUS12_4) of circUS12 by sequencing, the products of which were unvisualized due to their lower abundances. By calculation, circUS12_3 was predicted to be 285nt in length, while circUS12_4 was 799nt in length (**Figure 3B**). The full length of circUS12_3 was obtained by “rolling replication,” while part of circUS12_4 was gained and verified by Sanger sequencing. The sequence of circUS12_4 is composed of most of the US12 transcript and parts of the intronic regions between US12 and US13.

### HCMV circUL55 possesses two isoforms during lytic infection

**Figure 4:**
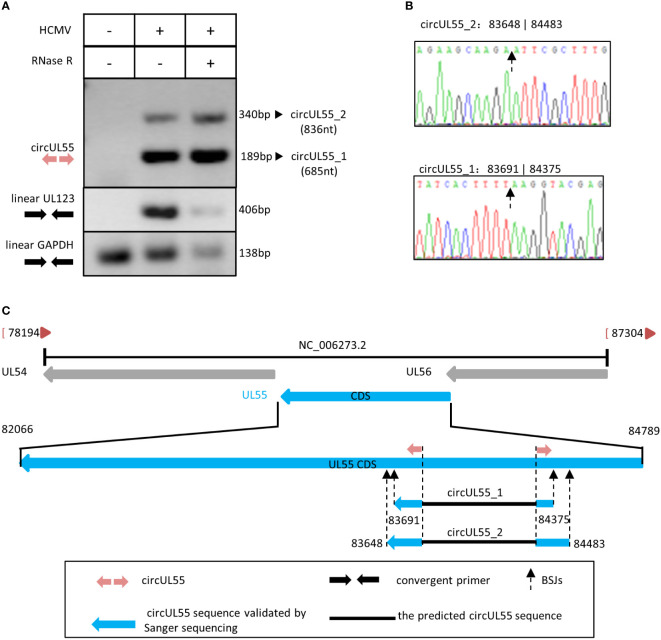
Validation and characterization of HCMV circUL55. **(A)** Inverse RT-PCR products of circUL55 with BSJs validation primer set in **Figure 1**. The 189 bp band that amplified from circUL55_1 (685 nt) was the predicted amplicon of the primer. A 340 bp band from circUL55_2 (836 nt) was amplified. Linear GAPDH and linear UL123 were amplified as RNase R-resistant control. **(B)** Sanger sequencing result of circUL55_1 and circUL55_2 BSJs. **(C)** Isoforms of circUL55mapping to HCMV UL55 gene.

Conducting RT-PCR by circUL55 divergent primers, we got two clean products containing circUL55 BSJ sequences that did not influence by RNase R. At the same time, linear UL123 and GAPDH amplicons were diminished apparently by RNase R (**Figure 4A**). Therefore, it was indicated that the two amplicons amplified by circUL55 divergent primer are authentic circular RNA.

It also suggested that circUL55 might have two isoforms, circUL55_1 and circUL55_2, during lytic infection (**Figure 4B**). CircUL55_1 was predicted to be 685nt in size. The validated BSJ junction of circUL55_1 was consistent with our RNA-seq results. CircUL55_2 presented a lower abundance than circUL55_1 and was predicted to be an 836nt circular transcription. By comparison, it was found that the splicing site of circUL55_2 was close to that of circUL55_1 (**Figure 4C**). RT-PCR and Sanger sequencing uncovered only part of the sequence in the two kinds of circUL55 isoforms. To achieve the whole sequence of these two circUL55, we designed the outer sequence amplify primer, which are convergent primers, based on the flank sequence around the circUL55 BSJ. Unfortunately, we could not amplify the outer sequence of the circUL55. The entire sequence might be achieved by the rolling replication mentioned above in future studies.

### HCMV circUL89 was 3890nt in length without splicing

**Figure 5:**
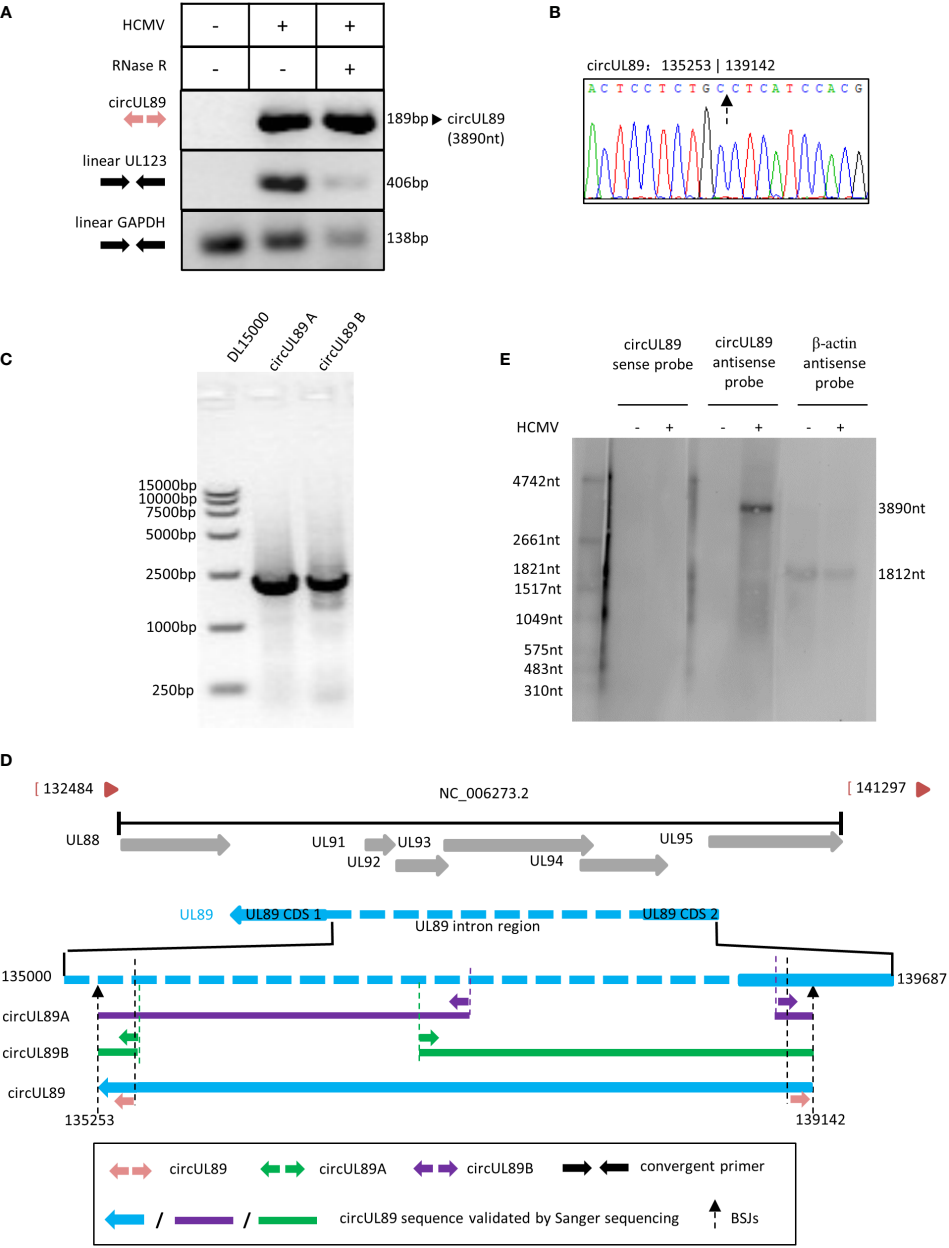
Validation and characterization of HCMV circUL89. **(A)** Inverse RT-PCR products of circUL55 with BSJs validation primer set in **Figure 1**. A 189 bp band was achieved as antipicated. Linear GAPDH and linear UL123 were amplified as RNase R-resistant control. **(B)** Sanger sequencing result of circUL89 BSJ. **(C)** Inverse RT-PCR results of HCMV circUL89 fragments A and B with divergent outer primer sets indicated in **Figure 1**. Both of the two fragments, which were overlapped in two terminals, were about 2000 bp in size. The whole sequence of circUL89 was achieved by sequencing and splicing. **(D)** The circUL89 full sequence mapping to HCMV UL89 gene. **(E)** Northern blot of circUL89.

Based on our RNA sequencing results, circUL89 was predicted to be a long circRNA with 3890nt in length. The potential circUL89 junction site sequence was amplified using divergent primers, whose products with the expected size of 189nt. As for circUL55 and circUS12, we used linear UL123 and linear GAPDH as the control for the RNase R treated assay. The results demonstrated that the circUL89 RNA was resistant to RNase R digestion, while linear UL123 and GAPDH diminished. It suggested that the sequence amplified by circUL89 divergent primers was the junction site of circUL89, and circUL89 was an existed viral circular RNA (**Figure 5A**). By cloning and sequencing, the sequence was confirmed to be the BSJ region of circUL89 (**Figure 5B**). It was indicated that HCMV circUL89 possessed a single transcript without alternative splicing.

Linear UL89 mRNA has coding sequences (CDS) generated by splicing. To address whether circUL89 was produced from existing linear mRNA or primary RNA independently, we designed two more divergent primer pairs (circUL89A and circUL89B) overlapping in products’ terminals to acquire the entire sequence of circUL89. Theoretically, the divergent primer circUL89A and circUL89B amplify a product ~2000nt in length, respectively (**Figure 1B**). As our anticipation, we amplified a prominent band with these two primer sets, respectively (**Figure 5C**). Then these two PCR products were subcloned in pcR2.1 vector, respectively. Sanger sequencing was performed to achieve the whole sequence of circUL89 (**Figure 5D**). After ligation of sequences of the products, we got the entire circUL89 sequence. Northern blots were applied to detect the transcriptions of circUL89 in mock- and HCMV-infected cells. It has been determined that linear UL89 mRNA is an antisense transcript in HCMV genome. The results showed that circUL89 was also an antisense transcript and derived from the negative-stranded DNA (**Figure 5E**). Unlike linear UL89 mRNA, it was confirmed that circUL89 was 3890nt in length as predicted without splicing, which consisted of the second CDS and part of the intron sequences of linear UL89 mRNA.

### HCMV circRNAs were predicted to target cellular and viral miRNAs

It’s well established that circRNA can influence miRNA functions by serving as miRNA sponges (Kristensen et al., 2019). Therefore, to gain insights into the functions of HCMV-encoded circRNAs, putative target miRNAs of dominant circUS12, circUL89 and circUL55 were predicted using miRanda software.

**Figure 6:**
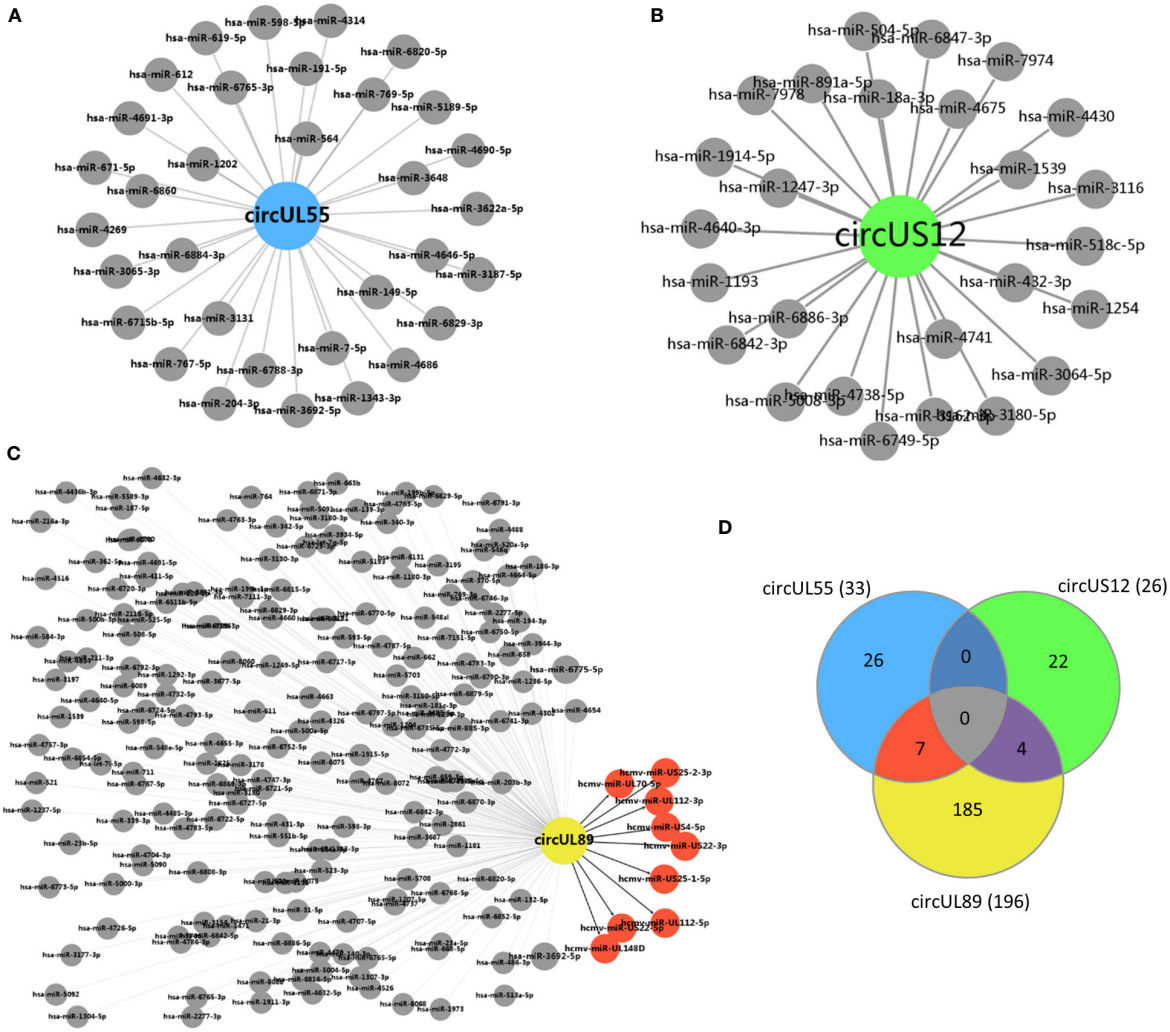
Prediction of putative target miRNAs of HCMV circUS12, circUL55, and circ UL89. **(A–C)** Prediction of putative target miRNAs of HCMV circUS12, circUL55, and circUL89. The putative cellular miRNAs of HCMV circUS12, circUL55, and circUL89 were 26, 33, and 196, respectively. Especially, nine HCMV-derived miRNAs were predicted to be the target of circUL89. **(D)** Venn diagram showing the number of unique or shared target miRNAs of circUS12, circUL55, and circUL89.

A total number of 26 cellular miRNAs were predicted to be targets of circUS12 (**Figure 6A**). The target genes of these 26 miRNAs were further predicted and analyzed for their putative functions. Results of GO analysis indicated that the target genes were involved in multiple important functions, including ion binding, enzyme/protein binding, and transcription factor activity (**Table 2**). Additionally, 33 and 196 cellular miRNAs were predicted to be targeted by circUL55 and circUL89, respectively (**Figures 6B, C**). There were shared target miRNAs among circUS12, circUL55, and circUL89 (**Figure 6D**).

**Table 2:** Putative molecular functions of target host miRNAs of HCMV circUS12.

Molecular_function	*p*-Value	Number of target genes	Number of hsa-miRNA
ion binding	9.74E-40	1190	26
nucleic acid binding transcription factor activity	2.44E-09	207	26
enzyme binding	1.08E-07	254	26
cytoskeletal protein binding	0.005217	144	26
protein binding transcription factor activity	0.047217	85	24

HCMV has been known to encode mature miRNAs. Only circUL89 was predicted to binding with viral miRNAs. As shown in **Figure 6C**, nine viral miRNAs (hcmv-miR-UL112-5p, hcmv-miR-UL112-3p, hcmv-miR-UL148D, hcmv-miR-US25-1-5p, hcmv-miR-US25-2-3p, hcmv-miR-US4-5p, hcmv-miR-UL70-5p, hcmv-miR-US22-5p and hcmv-miR-US22-3p) were predicted to bind with circUL89. Previous studies have validated a series of target genes of these viral miRNAs, which participate in many processes of HCMV infection, including viral survival, immune evasion, and the establishment of latency (**Supplementary Table S2**).

## Discussion

HCMV has been known to produce ncRNAs, including miRNAs and long noncoding RNAs (lncRNAs), to cope with the host’s antiviral mechanism. For instance, RNA2.7, a lncRNA encoded by HCMV, was identified to serve as an anti-apoptosis factor and maintain high ATP levels in cells with lytic infection or inhibit RNA polymerase II serin-2 phosphorylation, thus resulting in the success of the viral replication cycle in host cells (Reeves et al., 2007; Huang et al., 2022).

As newly recognized ncRNAs, circRNAs are usually generated by back-splicing of exons, introns, or both (Memczak et al., 2013; Jeck and Sharpless, 2014; Liang and Wilusz, 2014; Starke et al., 2015). Benefiting from the missing cap structure and poly (A) tail in the circular formation, circRNAs are more resistant to exonuclease than their linear counterparts (Jin et al., 2016). More and more evidence discloses circRNAs’ vital functions in many biological processes, including transcriptional regulation and protein expression (Starke et al., 2015; Chen, 2016).

In recent years, some circRNAs encoded by herpesvirus have been identified and investigated in infected cells. Kaposi’s sarcoma herpesvirus (KSHV)-encoded circvIRF4 is found noticeably expressed in the KSHV-positive patient sample and infected primary vascular and lymphatic endothelial cells (Tagawa et al., 2021). CircBARTs produced by Epstein-Barr virus, are highly expressed in infected tissue and cell lines with potential functions in viral oncogenesis (Toptan et al., 2018). A newly published work by Yang et al. biomatically predicts HCMV-encoded circRNAs based on transcriptomic data from NCBI GEO database and validate viral circRNA expressions in cells infected by different clinical HCMV strains (TB40/E, Towne and Toledo, respectively) (Yang et al., 2022). Their results suggest that the expression patterns of HCMV circRNAs are different between different strains and cell lines. Thus, our investigation in viral circRNA profiles of HAN strain in HELFs would provide more evidences for understanding of HCMV circRNA expression characteristics.

In this study, a total number of 629 HCMV-encoded circRNAs were experimentally revealed in HELFs infected with HAN strain. These uncovered circRNAs noticed a novel transcription manner of the HCMV clinical strain genome. Similar to the change in the host circRNAs in HCMV-infected HELFs (Deng et al., 2021), alternative splicing was also observed in HCMV-encoded circRNAs. The occurrence of alternative splicing remarkably increases the diversity of possible transcripts in the HCMV transcriptome. It is well known that there are four kinds of circRNAs due to their locations: exonic, intronic, exon-intro, and intergenic RNA, respectively (Jeck et al., 2013; Zhang et al., 2013; Qu et al., 2015; Li et al., 2015b). Accordingly, it is found that 80% of HCMV-encoded circRNAs are exonic circRNAs, and the median length of which was about 500 nt in length, which was in accordance with previous reports (Dong et al., 2016). It seems that exonic circRNAs and size in 400~500 nt are the favorable factor in stabilizing the circRNAs (Chen and Sarnow, 1995; Jeck et al., 2013; Ashwal-Fluss et al., 2014).

The BSJ site information and the entire sequence of HCMV-encoded circUS12, circUL55, and circUL89 were then achieved, respectively. Some circRNA isoforms bearing BSJ different from RNA-seq were discovered in our validation. These circRNAs have lower abundance and might be circular byproducts of linear splicing (Kristensen et al., 2019). HCMV-encoded circUS12 and circUL55 were found to possess more than two isoforms. This alternative circularization (AC) phenomenon might account for the diversity of HCMV-encoded circRNAs. AC suggests that one single gene loci could generate circRNA isoforms somehow (Zhang et al., 2014). The existence of circRNA isoforms increases the complexity of HCMV transcriptomes and might provide hints of gene expression regulation of the HCMV genome.

The HCMV US12 gene belongs to the US12 gene family, including US12 to US21, which encode GPCR (G-protein-coupled receptor)-like proteins (Rigoutsos et al., 2003). Similar to GPCR, pUS12 is characteristic of the seven-transmembrane-domain (7TMD) in regions 65-75, 115-135, 145-167, 175-195, 200-220, 250-258, and 265-275 (Rigoutsos et al., 2003). pUS12 participates in the natural killer (NK) cell evasion by facilitating gpUL16, which affects NK cell recognition (Rölle et al., 2003; Fielding et al., 2017). In our study, four kinds of circular US12 transcripts were observed. CircUS12_1 and circUS12_2 were thought to be the main circUS12 isoform. Interestingly, circUS12_1 and circUS12_2 cover the 7TMD-encoding regions in the linear US12 transcript. It might remind us that NK cell activation may rescue by conversing linear US12 into circular US12 during infection. The presented study indicated that circular UL55 is proud to be formatted in the 3’ terminal, which encodes the cytoplasmic domain of the glycoprotein B (gB, gpUL55), a 906 amino acids containing protein encoded by HCMV UL55. The gB protein plays an essential role in virus entry, cell-to-cell spread, viral replication, and syncytium formation (Navarro et al., 1993; Isaacson and Compton, 2009). It is well established that gB comprises four structural regions: an ectodomain, a hydrophobic membrane-proximal region, a transmembrane domain, and a cytoplasmic domain (Pötzsch et al., 2011; Reuter et al., 2020). The ectodomain is the ideal target for vaccine design by bearing five antigenic domains (Schleiss, 2008; Reuter et al., 2020). Moreover, reachers manifested that C-tail terminus impaired cellular fusion during HCMV infection (Tugizov et al., 1995; Bold et al., 1996). Up to our data, we found that circular UL55 is proud to be formatted in the 3’ terminal, which encodes the cytoplasmic domain of the gB. The formation of circUL55 might alleviate gB function, especially the infected cells’ fusion and antigenic activity, which results in the escape of the host immunity.

CircUL89 was proved to be a circular RNA with 3890nt in length, which is longer than known HCMV-encoded circRNAs. HCMV UL89 gene has two CDS post-splicing, while no splicing was found for circUL89 by mapping to the genome. It is speculated that virus-encoded circRNA might be derived from primary RNA independent of the production of mRNA. pUL89 is a component of terminase, whose other components are pUL56 and pUL51. Terminase facilitates viral replication by genome packaging. Previous research suggested aspartate 463 and arginine 544 are vital domains for pUL89 (Theiß et al., 2019). Surprisingly, both of these domains are located on the second CDS of the UL89 transcript. Of course, the two domains also exist on the circUL89 transcript. This phenomenon arouses our reverie that the function of UL89 is down-regulated by producing circUL89, which benefits building stable infection in host cells.

CircRNAs could inhibit gene expression by serving as miRNA sponges (Hansen et al., 2013b; Hansen et al., 2013a). The putative binding miRNAs of HCMV circUS12, circUL55, and circUL89 were predicted in our study. The putative target miRNAs were illustrated for their known potential functions. The results revealed that, for circUS12, the functions of its putative target miRNAs were mainly involved in critical molecular processes, such as ion binding, enzyme/protein binding, and transcription factor activity. It suggests that HCMV-encoded circRNAs might play a role in regulating metabolic processes and gene transcription during infection. Besides host miRNAs, HCMV miRNAs were also predicted to be targets of circUL89. The putative target viral miRNAs of circUL89 were validated to be involved in immune evasion and establishing viral latency. Therefore, it was speculated that viral circRNAs might work as a regulator to keep homeostasis between immune clearance and immune evasion by sponging related miRNAs, which would avoid excessive consumption and benefit viral survival. As known, circRNAs always share miRNA response elements with linear mRNAs and/or lncRNAs for competition. Hence, in further research, confocal fluorescence *in situ* hybridization (FISH) with probes specific to miRNAs and to circRNA BSJs might be carried to distinguish miRNA interacted circRNAs from linear mRNAs and/or lncRNAs.

The sequence of HCMV-encoded circUS12 and UL55 were identical to their linear counterparts. Some circRNAs have been discovered to be able to produce protein products (Legnini et al., 2017; Pamudurti et al., 2017). HCMV pUL55 is a fusion protein and an essential target of the host immune system (Paradowska et al., 2015). Whether HCMV circUS12 and circUL55 could produce proteins homologous to their host genes, it might be a method for HCMV proteins to escape from clearance at transcriptional levels.

In summary, this study experimentally delineated the profile of HCMV-encoded circRNAs in HELFs with lytic infection, laying a primary foundation for further elucidating the mechanisms of HCMV pathogenesis. However, more research is needed to investigate the functions of HCMV-encoded circRNAs further.

## Data availability statement

The datasets presented in this study can be found in online repositories. The names of the repository/repositories and accession number(s) can be found in the article/**Supplementary Material**.

## Author contributions

JD, YH, and QR conceived and designed the experiments. JD, JZ, QW, YL, and ZL performed the experiments. YM and YQ analyzed the data. JD wrote the manuscript and prepared the figures. QR and YH checked and finalized the manuscript. All authors contributed to the article and approved the final manuscript.

## Funding

This work was supported by the National Natural Science Foundation of China under Grant No. 82071664.

## Conflict of interest

The authors declare that the research was conducted in the absence of any commercial or financial relationships that could be construed as a potential conflict of interest.

## Publisher’s note

All claims expressed in this article are solely those of the authors and do not necessarily represent those of their affiliated organizations, or those of the publisher, the editors and the reviewers. Any product that may be evaluated in this article, or claim that may be made by its manufacturer, is not guaranteed or endorsed by the publisher.

## Supplementary material

Supplementary Figure 1 Morphology of mock-treated and HCMV-infected HELFs. HELFs were infected with HCMV at a multiplicity of infection (MOI) of 3. After 24 hours of incubation, the culture medium was replaced with MEM containing 2% FBS, 100 units/mL penicillin, and 100 μg/mL streptomycin. Meantime, PBS-treated HELFs were prepared as a mock-infected control. The HCMV-infected and mock-infected HELFs were harvested at 72 hours post infection (hpi).
