# Supplementary figures and images for: Identification and characterization of human cytomegalovirus-encoded circular RNAs
Source: Front Cell Infect Microbiol. 2022 Nov 14;12:980974. doi: 10.3389/fcimb.2022.980974 (PMC9702070; doi:10.3389/fcimb.2022.980974)

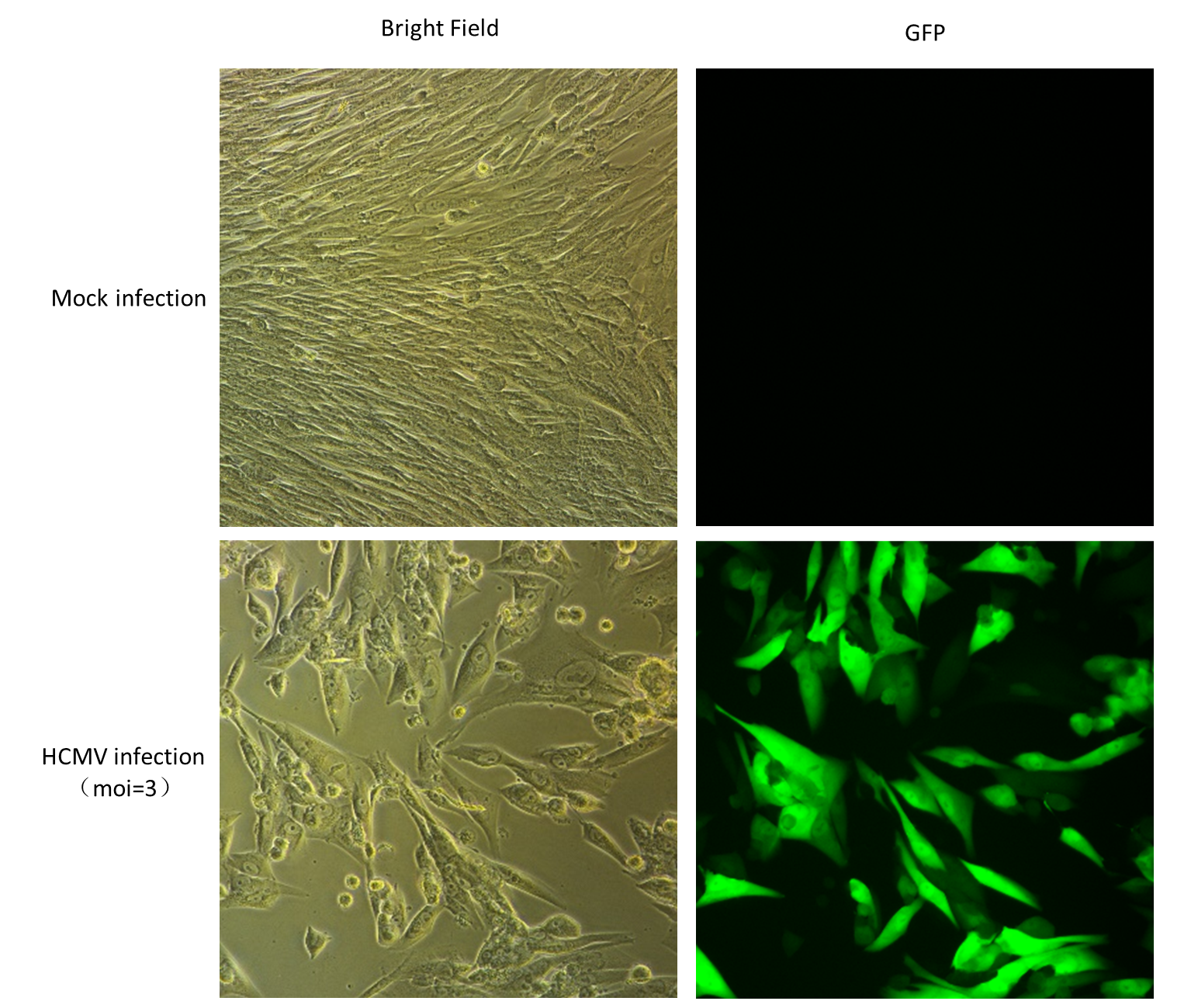

Supplement: Supplementary Figure 1 — Morphology of mock-treated and HCMV-infected HELFs. HELFs were infected with HCMV at a multiplicity of infection (MOI) of 3. After 24 hours of incubation, the culture medium was replaced with MEM containing 2% FBS, 100 units/mL penicillin, and 100 μg/mL streptomycin. Meantime, PBS-treated HELFs were prepared as a mock-infected control. The HCMV-infected and mock-infected HELFs were harvested at 72 hours post infection (hpi). [file Image_1.tif]
