# Supplementary material for: Identification and characterization of human cytomegalovirus-encoded circular RNAs
Source: Front Cell Infect Microbiol. 2022 Nov 14;12:980974. doi: 10.3389/fcimb.2022.980974 (PMC9702070; doi:10.3389/fcimb.2022.980974)
Supplement: Supplementary file 2 [file Table_1.docx]

**Supplementary Table S1. Primers used in the present study**

| Gene | Primers | Sequence (5'→3') | Product |
| --- | --- | --- | --- |
| circUS12 | Forward | GCTCCAGAAGGGCAGTGTATCG | 226 bp |
|  | Reverse | CCTATGTCCTGTCATCCATGCTCTG |  |
| circUL55 | Forward | AAGTGGTGTGGATGTAAGCGTAGC | 189 bp |
|  | Reverse | TGAACGTGCGGACTCGGTGAT |  |
| circUL89 | Forward | CGATGGAGGTCTGCGAGAGGAA | 189 bp |
|  | Reverse | AGGGACGACAGGTGTTGCTTGT |  |
| circUL89A* | Forward | GGATGATGAACCAAGTCTTG | 2161 bp |
|  | Reverse | CGCCTTAATAATACAGCTACG |  |
| circUL89B** | Forward | CTACGATGACCACGAAGTC | 2234 bp |
|  | Reverse | CAGGTGTTGCTTGTCTCC |  |
| UL123 | Forward | CATCCACATCTCCCGCTTAT | 406 bp |
|  | Reverse | GCACCGTCAAGGCTGAGAAC |  |
| GAPDH | Forward | TGGTGAAGACGCCAGTGGA | 138 bp |
|  | Reverse | GCACCGTCAAGGCTGAGAAC |  |
| circUL89 sense probe | Forward | TAATACGACTCACTATAGGGGCGTAGAGCG  AGTGTAAC | 429 bp |
|  | Reverse | ATGCCGATCATGTGCTTAA |  |
| circUL89 antisense probe | Forward | GCGTAGAGCGAGTGTAAC | 429 bp |
|  | Reverse | TAATACGACTCACTATAGGGATGCCGATCATGTGCTTAA |  |
| β-actin antisense probe | Forward | CTCTTCCAGCCTTCCTTC | 376 bp |
|  | Reverse | TAATACGACTCACTATAGGGGGTGTAACGCAACTAAGTC |  |

*circUL89A, primer set used to amplify circUL89 fragment A; **circUL89, primer set used to amplify circUL89 fragment A fragment B; bp, base pairs
