# Supplementary material for: Identification and characterization of human cytomegalovirus-encoded circular RNAs
Source: Front Cell Infect Microbiol. 2022 Nov 14;12:980974. doi: 10.3389/fcimb.2022.980974 (PMC9702070; doi:10.3389/fcimb.2022.980974)
Supplement: Supplementary file 3 [file Table_2.docx]

**Supplementary Table S2. Summary of functions of circUL89 putative target HCMV miRNAs**

| HCMV encoded microRNAs | Targets | Potential functions | Reference |
| --- | --- | --- | --- |
| miR-UL112 | IE72 (UL123, IE1), UL112/113, UL120/121 | Viral infection | (Grey et al., 2007) |
|  | UL114 | Viral infection | (Stern-Ginossar et al., 2009) |
|  | IL-32 | Immune evasion | (Huang et al., 2013) |
|  | VAMP3, RAB5C, RAB11A, SNAP23, CDC42 | Vesicle pathway | (Hook et al., 2014) |
|  |  | Viral latent infection | (Fu et al., 2014) |
|  | type I IFN signaling | Immune evasion | (Huang et al., 2015) |
|  | IE72 | Immune evasion | (Lau et al., 2016b) |
|  | MAPK signaling | Cell survival | (Shen et al., 2018) |
| miR-UL112-5p | ERAP1 | Immune evasion | (Romania et al., 2017) |
| miR-UL112-3p | MICB | Immune evasion | (Stern-Ginossar et al., 2007) |
|  | IRF-1,MICB | Immune evasion | (Li et al., 2011) |
|  | TLR2 | Immune evasion | (Landais et al., 2015) |
|  | IKKa, IKKb | Immune evasion | (Hancock et al., 2017) |
|  | TUSC3 | Cell survival | (Liang et al., 2017) |
|  | FOXO3a | Viral latent infection | (Hancock et al., 2021) |
| miR-US25-1 |  | Viral infection | (Stern-Ginossar et al., 2009) |
|  | CCNE2, BRCC3, EID1, MAPRE2, CD147 | Cell survival | (Grey et al., 2010) |
|  | BRCC3 | Cell survival | (Fan et al., 2014) |
|  |  | Viral latent infection | (Fu et al., 2014) |
|  | RhoA | Viral latent infection | (Diggins et al., 2021) |
| miR-US25-1-5p | YWHAE, UBB, NPM1, HSP90AA1 | Viral infection | (Jiang et al., 2015) |
|  | CD147 | Viral lytic propagation | (Chen et al., 2017) |
| miR-US22 | EGR-1 | Viral latency, viral reactivation | (Mikell et al., 2019) |
|  | SMAD3 | viral latency and reactivation | (Hancock et al., 2020) |
| miR-US4-1 | ERAP1 | Immune evasion | (Kim et al., 2011) |
|  | QARS | Cell survival | (Shao et al., 2016) |
| miR-UL148D | CCL5 | Immune evasion | (Kim et al., 2012) |
|  | IEX-1 | Cell survival | (Wang et al., 2013) |
|  | CDC25B | Latent infection | (Pan et al., 2016) |
|  | ACVR1B | Immune evasion | (Lau et al., 2016a) |

CHEN, J., XIA, S., YANG, X., CHEN, H., LI, F., LIU, F. & CHEN, Z. 2017. Human Cytomegalovirus Encoded miR-US25-1-5p Attenuates CD147/EMMPRIN-Mediated Early Antiviral Response. *Viruses,* 9.

DIGGINS, N. L., CRAWFORD, L. B., HANCOCK, M. H., MITCHELL, J. & NELSON, J. A. 2021. Human Cytomegalovirus miR-US25-1 Targets the GTPase RhoA To Inhibit CD34(+) Hematopoietic Progenitor Cell Proliferation To Maintain the Latent Viral Genome. *mBio,* 12.

FAN, J., ZHANG, W. & LIU, Q. 2014. Human cytomegalovirus-encoded miR-US25-1 aggravates the oxidised low density lipoprotein-induced apoptosis of endothelial cells. *Biomed Res Int,* 2014**,** 531979.

FU, M., GAO, Y., ZHOU, Q., ZHANG, Q., PENG, Y., TIAN, K., WANG, J. & ZHENG, X. 2014. Human cytomegalovirus latent infection alters the expression of cellular and viral microRNA. *Gene,* 536**,** 272-8.

GREY, F., MEYERS, H., WHITE, E. A., SPECTOR, D. H. & NELSON, J. 2007. A human cytomegalovirus-encoded microRNA regulates expression of multiple viral genes involved in replication. *PLoS Pathog,* 3**,** e163.

GREY, F., TIRABASSI, R., MEYERS, H., WU, G., MCWEENEY, S., HOOK, L. & NELSON, J. A. 2010. A viral microRNA down-regulates multiple cell cycle genes through mRNA 5'UTRs. *PLoS Pathog,* 6**,** e1000967.

HANCOCK, M. H., CRAWFORD, L. B., PEREZ, W., STRUTHERS, H. M., MITCHELL, J. & CAPOSIO, P. 2021. Human Cytomegalovirus UL7, miR-US5-1, and miR-UL112-3p Inactivation of FOXO3a Protects CD34(+) Hematopoietic Progenitor Cells from Apoptosis. *mSphere,* 6.

HANCOCK, M. H., CRAWFORD, L. B., PHAM, A. H., MITCHELL, J., STRUTHERS, H. M., YUROCHKO, A. D., CAPOSIO, P. & NELSON, J. A. 2020. Human Cytomegalovirus miRNAs Regulate TGF-β to Mediate Myelosuppression while Maintaining Viral Latency in CD34(+) Hematopoietic Progenitor Cells. *Cell Host Microbe,* 27**,** 104-114.e4.

HANCOCK, M. H., HOOK, L. M., MITCHELL, J. & NELSON, J. A. 2017. Human Cytomegalovirus MicroRNAs miR-US5-1 and miR-UL112-3p Block Proinflammatory Cytokine Production in Response to NF-κB-Activating Factors through Direct Downregulation of IKKα and IKKβ. *mBio,* 8.

HOOK, L. M., GREY, F., GRABSKI, R., TIRABASSI, R., DOYLE, T., HANCOCK, M., LANDAIS, I., JENG, S., MCWEENEY, S., BRITT, W. & NELSON, J. A. 2014. Cytomegalovirus miRNAs target secretory pathway genes to facilitate formation of the virion assembly compartment and reduce cytokine secretion. *Cell Host Microbe,* 15**,** 363-73.

HUANG, Y., CHEN, D., HE, J., CAI, J., SHEN, K., LIU, X., YANG, X. & XU, L. 2015. Hcmv-miR-UL112 attenuates NK cell activity by inhibition type I interferon secretion. *Immunol Lett,* 163**,** 151-6.

HUANG, Y., QI, Y., MA, Y., HE, R., JI, Y., SUN, Z. & RUAN, Q. 2013. The expression of interleukin-32 is activated by human cytomegalovirus infection and down regulated by hcmv-miR-UL112-1. *Virol J,* 10**,** 51.

JIANG, S., QI, Y., HE, R., HUANG, Y., LIU, Z., MA, Y., GUO, X., SHAO, Y., SUN, Z. & RUAN, Q. 2015. Human cytomegalovirus microRNA miR-US25-1-5p inhibits viral replication by targeting multiple cellular genes during infection. *Gene,* 570**,** 108-14.

KIM, S., LEE, S., SHIN, J., KIM, Y., EVNOUCHIDOU, I., KIM, D., KIM, Y. K., KIM, Y. E., AHN, J. H., RIDDELL, S. R., STRATIKOS, E., KIM, V. N. & AHN, K. 2011. Human cytomegalovirus microRNA miR-US4-1 inhibits CD8(+) T cell responses by targeting the aminopeptidase ERAP1. *Nat Immunol,* 12**,** 984-91.

KIM, Y., LEE, S., KIM, S., KIM, D., AHN, J. H. & AHN, K. 2012. Human cytomegalovirus clinical strain-specific microRNA miR-UL148D targets the human chemokine RANTES during infection. *PLoS Pathog,* 8**,** e1002577.

LANDAIS, I., PELTON, C., STREBLOW, D., DEFILIPPIS, V., MCWEENEY, S. & NELSON, J. A. 2015. Human Cytomegalovirus miR-UL112-3p Targets TLR2 and Modulates the TLR2/IRAK1/NFκB Signaling Pathway. *PLoS Pathog,* 11**,** e1004881.

LAU, B., POOLE, E., KRISHNA, B., SELLART, I., WILLS, M. R., MURPHY, E. & SINCLAIR, J. 2016a. The Expression of Human Cytomegalovirus MicroRNA MiR-UL148D during Latent Infection in Primary Myeloid Cells Inhibits Activin A-triggered Secretion of IL-6. *Sci Rep,* 6**,** 31205.

LAU, B., POOLE, E., VAN DAMME, E., BUNKENS, L., SOWASH, M., KING, H., MURPHY, E., WILLS, M., VAN LOOCK, M. & SINCLAIR, J. 2016b. Human cytomegalovirus miR-UL112-1 promotes the down-regulation of viral immediate early-gene expression during latency to prevent T-cell recognition of latently infected cells. *J Gen Virol,* 97**,** 2387-2398.

LI, S., ZHU, J., ZHANG, W., CHEN, Y., ZHANG, K., POPESCU, L. M., MA, X., LAU, W. B., RONG, R., YU, X., WANG, B., LI, Y., XIAO, C., ZHANG, M., WANG, S., YU, L., CHEN, A. F., YANG, X. & CAI, J. 2011. Signature microRNA expression profile of essential hypertension and its novel link to human cytomegalovirus infection. *Circulation,* 124**,** 175-84.

LIANG, Q., WANG, K., WANG, B. & CAI, Q. 2017. HCMV-encoded miR-UL112-3p promotes glioblastoma progression via tumour suppressor candidate 3. *Sci Rep,* 7**,** 44705.

MIKELL, I., CRAWFORD, L. B., HANCOCK, M. H., MITCHELL, J., BUEHLER, J., GOODRUM, F. & NELSON, J. A. 2019. HCMV miR-US22 down-regulation of EGR-1 regulates CD34+ hematopoietic progenitor cell proliferation and viral reactivation. *PLoS Pathog,* 15**,** e1007854.

PAN, C., ZHU, D., WANG, Y., LI, L., LI, D., LIU, F., ZHANG, C. Y. & ZEN, K. 2016. Human Cytomegalovirus miR-UL148D Facilitates Latent Viral Infection by Targeting Host Cell Immediate Early Response Gene 5. *PLoS Pathog,* 12**,** e1006007.

ROMANIA, P., CIFALDI, L., PIGNOLONI, B., STARC, N., D'ALICANDRO, V., MELAIU, O., LI PIRA, G., GIORDA, E., CARROZZO, R., BERGVALL, M., BERGSTRöM, T., ALFREDSSON, L., OLSSON, T., KOCKUM, I., SEPPäLä, I., LEHTIMäKI, T., HURME, M. A., HENGEL, H., SANTONI, A., CERBONI, C., LOCATELLI, F., D'AMATO, M. & FRUCI, D. 2017. Identification of a Genetic Variation in ERAP1 Aminopeptidase that Prevents Human Cytomegalovirus miR-UL112-5p-Mediated Immunoevasion. *Cell Rep,* 20**,** 846-853.

SHAO, Y., QI, Y., HUANG, Y., LIU, Z., MA, Y., GUO, X., JIANG, S., SUN, Z. & RUAN, Q. 2016. Human cytomegalovirus-encoded miR-US4-1 promotes cell apoptosis and benefits discharge of infectious virus particles by targeting QARS. *J Biosci,* 41**,** 183-92.

SHEN, K., XU, L., CHEN, D., TANG, W. & HUANG, Y. 2018. Human cytomegalovirus-encoded miR-UL112 contributes to HCMV-mediated vascular diseases by inducing vascular endothelial cell dysfunction. *Virus Genes,* 54**,** 172-181.

STERN-GINOSSAR, N., ELEFANT, N., ZIMMERMANN, A., WOLF, D. G., SALEH, N., BITON, M., HORWITZ, E., PROKOCIMER, Z., PRICHARD, M., HAHN, G., GOLDMAN-WOHL, D., GREENFIELD, C., YAGEL, S., HENGEL, H., ALTUVIA, Y., MARGALIT, H. & MANDELBOIM, O. 2007. Host immune system gene targeting by a viral miRNA. *Science,* 317**,** 376-81.

STERN-GINOSSAR, N., SALEH, N., GOLDBERG, M. D., PRICHARD, M., WOLF, D. G. & MANDELBOIM, O. 2009. Analysis of human cytomegalovirus-encoded microRNA activity during infection. *J Virol,* 83**,** 10684-93.

WANG, Y. P., QI, Y., HUANG, Y. J., QI, M. L., MA, Y. P., HE, R., JI, Y. H., SUN, Z. R. & RUAN, Q. 2013. Identification of immediate early gene X-1 as a cellular target gene of hcmv-mir-UL148D. *Int J Mol Med,* 31**,** 959-66.
